# Type 1 Diabetes Is a Barrier to Obesity Treatment: Patient Insights From a Mixed‐Methods Study

**DOI:** 10.1155/jobe/1461796

**Published:** 2025-12-14

**Authors:** Ebaa Al Ozairi, Dalal Alsaeed, Alvin Mondoh, Etab Taghadom, Mohammad Irshad, Dherar Alroudhan, Jumana Al Kandari, Werd Al-Najim, Carel W. le Roux

**Affiliations:** ^1^ DAFNE Unit, Clinical Care Research and Clinical Trials Unit, Dasman Diabetes Institute, Kuwait City, Kuwait, dasmaninstitute.org; ^2^ Diabetes Complications Research Centre, University College Dublin, Dublin, Ireland, ucd.ie; ^3^ Amiri Hospital, Kuwait City, Kuwait, amirihospital.com; ^4^ Diabetes Research Centre, Ulster University, Coleraine, UK, ulster.ac.uk

**Keywords:** body weight perceptions, obesity, T1D

## Abstract

**Background:**

Globally, many patients with Type 1 diabetes (T1D) are now characterized by excess adipose tissue and features of insulin resistance. In Kuwait, rapid urbanization, shifts in dietary patterns, and decreased physical activity have contributed to rising obesity prevalence in the general population.

**Objectives:**

We aimed to investigate the interplay between the diseases of obesity and T1D, examining patients’ perspectives on why they gained body weight, psychological aspects, and management challenges.

**Methods:**

A mixed‐methods approach was employed, encompassing quantitative analysis of body mass index (BMI) and lifestyle factors among 51 participants with T1D and obesity or obesity‐related complications and a thematic analysis of perceptions and experiences related to obesity and T1D using an online survey.

**Results:**

Participants identified lifestyle factors as the primary contributors to obesity, emphasizing the need for holistic interventions. About 56.8% of the participants perceived T1D as a barrier to obesity treatment. The qualitative analysis revealed four themes: 1) negative perceptions about obesity, 2) poor interface with healthcare professionals (HCPs), 3) lack of suggestions for improving obesity management, and 4) poor self‐image and awareness. This provided in‐depth insights into participants’ perceptions, worries, experiences, and suggestions for managing obesity in the context of T1D.

**Conclusions:**

This study contributes a nuanced understanding of obesity in patients with T1D, shedding light on the complexities beyond glycemic control. The findings emphasize the need for patient‐centered, multidisciplinary approaches that consider both medical and psychological aspects in the management of obesity within patients with T1D.

## 1. Introduction

Obesity, as defined by the World Health Organization (WHO) as excess adipose tissue causing a deterioration in health [1], has not traditionally been associated with T1D [2]. Rather, T1D was associated with lower body weight due to insulin deficiency [1, 2]. T1D is an autoimmune disease that destroys pancreatic beta cells, resulting in a lifelong reliance on insulin supplementation. Conversely, Type 2 diabetes (T2D) is marked by insulin resistance and defective insulin secretion [2, 3].

Previously, T1D was associated with underweight status and catabolic metabolism. However, this has profoundly evolved over the past 2 decades as there has been a global surge in overweight and obesity among individuals with T1D, leading to the emergence of “double diabetes” [4, 5]. Globally, many patients with T1D are now characterized by excess adipose tissue, features of insulin resistance, and a family history of T2D [4]. This trend is particularly evident in the Middle East, a region with some of the highest global rates of T1D [6, 7]. In Kuwait, rapid urbanization, shifts in dietary patterns, and decreased physical activity have contributed to rising obesity prevalence in the general population [8]. However, limited research has only examined the impact of obesity on individuals with T1D in this context.

Recent epidemiological and clinical studies have challenged the traditional distinction between T1D and T2D, demonstrating that both conditions share underlying mechanisms, including inflammation associated with excess adiposity, insulin resistance, and dyslipidemia [3, 9]. In individuals with T1D, obesity not only contributes to glycemic instability but also increases the risk of complications, including retinopathy, nephropathy, neuropathy, and cardiovascular disease [10]. Despite these risks, obesity in T1D remains frequently under‐recognized and inadequately addressed, as clinical management often prioritizes glycemic control [11]. Simultaneously, epidemiological trends indicate a shift, with an increasing number of patients with T1D being diagnosed with the disease of obesity [12, 13]. Obesity in people with T1D has been associated with environmental factors, high‐energy density diets, sedentary lifestyles, altered eating behaviors, genetic predispositions, and psychosocial factors [14]. It also increases with age and diabetes duration [13].

The intricate interplay between T1D and obesity poses challenges to disease management, compounded by a notable absence of well‐defined treatment strategies [15, 16]. Obesity in people with T1D increases the risk of microvascular complications, including retinopathy, nephropathy, and neuropathy [10]. It also elevates cardiovascular risk factors, such as hypertension and dyslipidemia [9]. Nevertheless, a previous pilot study demonstrated that people with obesity and T1D exhibited uncontrolled glycemic levels nearly twice compared to those with normal body mass index (BMI) and T1D [13].

Recent studies demonstrate that obesity in individuals with T1D represents both a metabolic comorbidity and a psychosocial and behavioral challenge [17, 18]. Individuals with T1D frequently experience a conflict between the necessary administration of insulin and its propensity to induce weight gain. This conflict adversely affects body image, self‐perception, and adherence to prescribed treatment regimens [5]. Furthermore, the simultaneous management of T1D and obesity can exacerbate psychological distress, increase perceived stigma, and contribute to therapeutic fatigue [8, 16].

Obesity in people with T1D transcends conventional glycemic control concerns, extending its impact to encompass heightened insulin resistance, elevated cardiovascular risks, and a broader influence on overall well‐being. Moreover, the psychosocial dimensions of body weight perceptions and the experiences of being diagnosed with obesity introduce layers of complexity often overlooked in existing scientific literature. Although awareness of these challenges is growing, few empirical studies investigate how individuals with T1D perceive obesity, understand its causes, and interact with the healthcare system. Most existing approaches prioritize clinician perspectives and overlook patient input, which could enhance the development of more responsive and patient‐centered care models [19, 20]. Recognizing the multifaceted nature of this dual challenge, there is a shift toward a more patient‐centered care approach [20]. Emphasizing patient experiences, perceptions, and individualized needs becomes paramount in tailoring effective interventions that address the intersections of T1D and obesity. This paradigm shift is essential for optimizing care and support, ensuring a holistic approach that accounts for this unique population’s physiological and psychological dimensions [19, 20].

We aimed to explore the interplay between body weight perceptions, contributing factors, and managing obesity in people with T1D with the disease of obesity. By integrating quantitative data on BMI and lifestyle factors with qualitative insights into participants’ perceptions and experiences, we sought to provide a comprehensive understanding, offering insights to optimize care and support to patients with T1D and obesity.

## 2. Materials and Methods

### 2.1. Study Design

A mixed‐methods approach was undertaken to achieve the study’s aim, incorporating both quantitative and qualitative analyses.

### 2.2. Study Setting

Participants were recruited from the Dose Adjustment for Normal Eating (DAFNE) register at the Dasman Diabetes Institute in Kuwait [8].

### 2.3. Study Population

People with T1D residing in Kuwait.

#### 2.3.1. Inclusion Criteria

All patients met the criteria for obesity as they had a BMI > 27 kg/m^2^ with obesity‐related complications or BMI > 30 kg/m^2^, and they were between ages 18 and 65 years.

### 2.4. Sampling Process, Sample Size, and Consent

Purposive sampling was used by selecting patients with T1D and a BMI ≥ 27 kg/m^2^ with obesity‐related complications or BMI > 30 kg/m^2^. The registry was filtered based on these criteria, and 300 patients were found eligible. For easier dissemination, the survey link was first sent to a sample of 60 patients from the registry. The survey was open for 2 weeks. The average time to complete the survey was 22 min. A consent statement was provided and signed by all participants at the start of the survey.

### 2.5. Data Collection

#### 2.5.1. Survey Development

A semistructured survey was developed using Microsoft Forms, following a review of the literature and refinement by the research team. The survey was made available in English and Arabic to accommodate all participants and consisted of a total of 40 questions, including demographics questions; the questions appeared in a sequential order based on the participant’s response, with some questions deemed not applicable due to nonapplicable earlier responses. The question format ranged from multiple choice to short answers. Topics included management of T1D, perceptions surrounding their weight and obesity, and experiences of obesity diagnosis and treatment. Participants were also provided with an opportunity at the end of the survey to share any thoughts on obesity treatments.

### 2.6. Data Analysis

Quantitative and qualitative analysis methods were utilized. The survey questionnaire was collected and reviewed, and the data were extrapolated and tabulated for analysis. All statistical analyses for this study were conducted using SPSS Version 29.0 software (IBM Corp.). Descriptive statistics were employed to measure proportions with percentages or means with their standard deviations for categorical and continuous variables, respectively. Pearson’s chi‐square test was utilized to evaluate and quantify associations between categorical outcomes. Further analyses were performed to estimate participants’ perceptions of contributory factors to obesity in terms of relative risk (RR) factors. A two‐sided *p*‐value ≤ 0.05 was considered statistically significant.

Open‐ended responses were extracted and analyzed thematically to gain an insight into participants’ perceptions surrounding obesity and its management in T1D. Initial coding was manually conducted by DA to develop a coding framework to categorize themes and subthemes. Thematic analysis results were discussed within the research group, and consensus was reached. Data saturation was determined when no new insights relevant to the research question emerged. Content analysis was also utilized to quantify participant results.

## 3. Results

### 3.1. Quantitative Results

Of the 51 respondents, 43 (84%) responded in Arabic and 8 (16%) responded in English. Table 1 presents the demographics of the 51 participants, with males comprising 52.9% and females 47.1%. Ages ranged from 21 to 69 years, and they were diagnosed with T1D at median age of 13 years (range 2–55 years). The mean BMI value was 33.1 ± 2.7 kg/m^2^ (range 27.1–46.8 kg/m^2^), and there was no significant difference in BMI between males and females (*p* = 0.88). All patients approached had a measured BMI > 30 kg/m^2^ in clinic, but only 45 of the participants (88.2%) had a self‐reported BMI > 30 kg/m^2^. Pump therapy was used in 14 participants (27.5%), and 37 participants (72.5%) were using multiple daily insulin injections. Twelve participants (23.5%) reported diabetes‐related complications, such as retinopathy, retinopathy, and neuropathy.

**Table 1:** Primary information of the participants.

		*n* (%) or mean (SD)
Sex	Female	24 (47.1)
	Male	27 (52.9)

Age (years)	21–29	7 (13.7)
	30–39	26 (51.0)
	40–49	13 (25.5)
	50–59	4 (7.8)
	60–69	1 (2.0)

Height	Meter	1.7 (0.1)
Weight	kg	91.8 (11.4)
BMI	kg/m^2^	33.1 (2.7)
Complications	No	39 (76.5)
	Yes	12 (23.5)

Insulin regimen	MDI	37 (72.5)
	Pump	14 (27.5)

Age at diagnosis	Years	16.2 (10.7)
Short‐acting insulin dose	U/day	37.6 (22.3)
Long‐acting insulin dose	U/day	28.7 (16.5)
Total daily dose	U/day	66.2 (32.5)

Abbreviations: MDI = multiple drug injection; SD = standard deviation.

#### 3.1.1. Perceptions

Forty‐six participants (92.2%) reported themselves as being overweight, while only 5 (9.8%) considered themselves as having obesity. Participants reported having a weight problem for 5.0 ± 3.4 years (range 0–30 years). Table 2 shows the association between participants’ self‐perceived body weight categories and their actual BMI. Despite having a BMI ≥ 30 kg/m^2^, 78.4% (*n* = 40) of the participants perceived themselves as not having obesity. A smaller percentage of participants considered themselves having obesity compared to their actual BMI of 30–34.9 kg/m^2^ in obesity class 1 (9.1% vs. 90.9%, *p* < 0.001). Even in those with an actual BMI ≥ 35 kg/m^2^, only 16.7% considered themselves having obesity compared with 83.3% who did not (*p* = 0.03).

**Table 2:** Actual BMI versus perceived weight categories of the participants.

Actual BMI		Perceived	*n* (%)	*X* ^2^ (*p*‐value)^∗^
> 27–30 kg/m^2^	6 (11.8)	Normal weight	1 (16.7)	2.7 (0.102)
		Overweight	5 (83.3)	

30–35 kg/m^2^	33 (64.7)	Overweight	30 (90.9)	22.1 (< 0.001)
		Living with obesity	3 (9.1)	

> 35 kg/m^2^	12 (23.5)	Overweight	10 (83.3)	5.3 (0.03)
		Living with obesity	2 (16.7)	

^∗^One‐way chi‐squared test.

In Table 3, the participants identified 10 contributing factors to them being overweight or having obesity. They reported that lifestyle factors had the highest RR compared to all other contributing factors (RR 2.1–12.0, *p* < 0.001). The side effects of medication emerged as the second‐highest RR factors when compared to physical environments, genetic predisposition, social/cultural pressure, negative early‐life experiences, insulin pump usage, onset of puberty, and pregnancy and childbirth (RR 2.4–5.7, *p* < 0.05). The third‐highest RR factor was a negative emotional state, in comparison with negative early‐life experiences, insulin pump usage, onset of puberty, and pregnancy and childbirth (RR 2.6–4.3, *p* < 0.05).

**Table 3:** Contributory factors for the development of overweight/obesity.

Contributors	N (%)	RR (95%CI)	RR (95%CI)	RR (95%CI)	RR (95%CI)	RR (95%CI)	RR (95%CI	RR (95%CI	RR (95%CI
Onset of puberty	3 5.9	1							
Pregnancy and childbirth	3 5.9	1							
Insulin pump	4 7.8	1.3 (0.3, 5.7)	1						
Negative early‐life experiences	5 9.8	1.7 (0.4, 6.6)	1.3 (0.4, 4.4)	1					
Societal/cultural pressures	6 11.8	2.0 (0.5, 7.6)	1.5 (0.4, 5.0)	1.2 (0.4, 3.7)	1				
Genetic disposition	7 13.7	2.3 (0.6, 8.5)	1.8 (0.5, 5.6)	1.4 (0.5, 4.1)	1.2 (0.4, 3.2)	1			
Physical environment	10 19.6	3.3 1.0, 11.4^∗^	2.5 (0.8, 7.5)	2.0 (0.7, 5.4)	1.7 (0.7, 4.3)	1.4 (0.6, 3.6)	1		
Negative emotion states	13 25.5	4.3 1.3, 14.3^∗^	3.3 1.1, 9.3^∗^	2.6 1.0, 6.8^∗^	2.2 (0.9, 5.3)	1.8 (0.8, 4.3)	1.3 (0.4796	1	
Side effect of medications	17 33.3	5.7 1.8, 18.2^∗∗^	4.3 1.5, 11.8^∗∗^	3.4 1.4, 8.5^∗∗^	2.8 1.2, 6.6^∗^	2.4 1.1, 5.4^∗^	1.7 0.9, 3.3	1.3 0.7, 2.4	1
Lifestyle	36 70.6	12.0 3.9, 36.5^∗∗^	9.0 3.6, 23.4^∗∗^	7.2 3.1, 16.9^∗∗^	6.0 2.8, 13.0^∗∗^	5.2 2.5, 10.5^∗∗^	3.6 2.0, 6.5^∗∗^	2.8 1.8, 4.6^∗∗^	2.1 1.4, 3.2^∗∗^

Abbreviations: CI = confidence interval, RR = relative risk.

^∗^ *p* < 0.05

^∗∗^ *p* < 0.001.

Table 4 illustrates the participants’ perceptions regarding obesity. The majority of participants believed that obesity is a disease (*n* = 43, 84.3%), and this perception was almost equally distributed among both sexes (*p* = 0.56). However, when asked about their personal experiences with being diagnosed with obesity, responses were varied. A significant portion reported negative experiences (41.2%, *n* = 21), while 25.5% (*n* = 13) stated that they had never had an official diagnosis, and 17.6% (*n* = 9) expressed that obesity “does not matter.”

**Table 4:** Participants’ perceptions about obesity.

		Total *n* (%)	Male *n* (%)	Female *n* (%)	*χ*2	*p*‐value
		51 (100)	27 (100)	24 (100)		
Obesity is disease	No	8 (15.7)	5 (18.5)	3 (12.5)	0.35	0.555
	Yes	43 (84.3)	22 (81.5)	21 (87.5)		

What is your experience of being diagnosed with overweight/obesity	Does not matter	9 (17.6)	6 (22.2)	3 (12.5)	1.45	0.693
	Negative	21 (41.2)	10 (37.0)	11 (45.8)		
	Never been officially diagnosed	13 (25.5)	6 (22.2)	7 (29.2)		
	Positive	8 (15.7)	5 (18.5)	3 (12.5)		

Have you ever sought treatment for your overweight/obesity?	No	14 (27.5)	8 (29.6)	6 (25.0)	0.14	0.712
	Yes	37 (72.5)	19 (70.4)	18 (75.0)		

Do you think obesity in T1D should be treated with	Lifestyle measures	26 (51.0)	16 (59.3)	10 (41.7)	3.41	0.332
	Lifestyle measures and medications	13 (25.5)	5 (18.5)	8 (33.3)		
	Lifestyle measures, medications, and surgery	8 (15.7)	5 (18.5)	3 (12.5)		
	Medications and surgery	4 (7.8)	1 (3.7)	3 (12.5)		

Have you ever been offered to see a dietician to help treat obesity?	No	7 (13.7)	5 (18.5)	2 (8.3)	1.11	0.291
	Yes	44 (86.3)	22 (81.5)	22 (91.7)		

Have you found dietary advice helpful for obesity management?	No	11 (21.6)	5 (18.5)	6 (25.0)	0.32	0.574
	Yes	40 (78.4)	22 (81.5)	18 (75.0)		

Do you know what the dietitian can advise/offer?	No	21 (41.2)	15 (55.6)	6 (25.0)	4.90	0.027
	Yes	30 (58.8)	12 (44.4)	18 (75.0)		

Do you think your T1D was a barrier to you receiving obesity treatment?	No	23 (45.1)	16 (59.3)	7 (29.2)	4.65	0.031
	Yes	28 (54.9)	11 (40.7)	17 (70.8)		

However, the majority sought treatment for their obesity (72.5%, *n* = 37). When questioned about the effectiveness of various weight loss options, most participants favored lifestyle measures (51.0%, *n* = 26), followed by a combination of lifestyle measures and medicine (25.5%, *n* = 13). Fewer participants supported the use of medicine alone or surgery as weight loss options (7.8%, *n* = 4).

A majority (86.3%, *n* = 44) confirmed that they sought guidance from a dietitian, and among them, 78.4% (*n* = 40) found the dietary advice helpful for managing obesity. In addition, a significant portion (58.8%, *n* = 30) indicated that they retained the dietitian’s advice for obesity management. Despite the advantages of dietitian advice, a larger proportion of females compared to males perceived T1D as a barrier to receiving obesity treatment (70.8% vs. 40.7%, *p* = 0.03).

The qualitative analysis in Table 5 provides key insights into participants’ perceptions and experiences regarding obesity in the context of T1D. A majority (56.9%) of respondents recognized obesity as a disease due to its association with other health risks, while a smaller portion (21.6%) linked it to physical appearance and mobility issues. The most common worries included increased blood glucose and insulin requirements (27.5%) and potential complications (21.6%). The diagnosis experience varied with 25.5% stating they had never been diagnosed, while others reported physical (9.8%) and mental health impacts (11.8%). Treatment‐seeking behavior showed mixed responses; 29.4% had been recommended medication, while 21.6% pursued a diet and exercise plan. Some (11.8%) opted for self‐management, and 5.9% did not seek treatment due to not perceiving themselves as having obesity. Regarding improving obesity treatment, participants emphasized the need for greater awareness (21.6%), encouragement of a healthy lifestyle (21.6%), and structured follow‐ups (15.7%). A small percentage (3.9%) suggested decreasing insulin as a potential intervention. These findings highlighted the complexity of obesity perception and management among individuals with T1D, underscoring the need for patient‐centered approaches that consider both medical and psychological factors.

**Table 5:** Qualitative analysis data.

Why do you think obesity may be a disease?	N (%)
Associated with risk of other diseases	29 (56.9)
Control by change in lifestyle	3 (5.9)
Need monitoring	1 (2.0)
Normal life	3 (5.9)
Not known	4 (7.8)
Physical appearance and movement	11 (21.6)

What worries you the most when you think about obesity with your T1D?	
Bone and joint problems	2 (3.9)
Complications	11 (21.6)
Fatigue	5 (9.8)
Hard to reduce weight	5 (9.8)
Heart disease	2 (3.9)
High blood glucose and insulin dose increase	14 (27.5)
Hypertension	2 (3.9)
Nothing	6 (11.8)
Vague answers	4 (7.8)

How would you describe your experience of being diagnosed with obesity?	
Affect my physical appearance and daily life	5 (9.8)
Does not matter	5 (9.8)
Fatigue/mental health issue	6 (11.8)
Hard to reduce weight	4 (7.8)
Never been diagnosed	13 (25.5)
Vulnerable to diseases	6 (11.8)
Weight reduction program	4 (7.8)
Willing to lose weight	8 (15.7)

Have you ever sought treatment for your obesity?	
Yes, recommended diet plan	2 (3.9)
Yes, recommended diet plan and exercise	11 (21.6)
Yes, recommended diet plan and medicine	2 (3.9)
Yes, recommended diet plan and surgery	3 (5.9)
Yes, recommended diet plan, medicine, and exercise	3 (5.9)
Yes, recommended medication	15 (29.4)
Yes, but not recommended	2 (3.9)
Not answered	4 (7.8)
No, because not suffering with obesity	3 (5.9)
No, because using self‐management plan	6 (11.8)

How can the process of treating obesity be improved?	
Awareness for obesity complications and healthy lifestyle	11 (21.6)
Decrease insulin	2 (3.9)
Encouragement of healthy lifestyle	11 (21.6)
Diet and exercise	6 (11.8)
Follow‐ups on healthy eating and exercise	8 (15.7)
No answer	7 (13.7)
Providing helpful medications	3 (5.9)
Psychologically	1 (2.0)
Special diet	1 (2.0)
Surgeries	1 (2.0)

### 3.2. Qualitative Results

The qualitative analysis yielded four major themes; the themes and subthemes are listed in Table 6 below.

**Table 6:** Themes and subthemes.

Theme	Subtheme
Perceptions about obesity	A disease or not a disease?
	Long‐term concerns
	Factors contributing to obesity development

Interface with HCPs	Diagnosis deficits
	Treatment options
	Role of dietitians

Suggestions for improving obesity management in T1D	Position diabetes within obesity management
	Focus on mental health
	Post–weight loss management
	Patient‐centered care
	Improve obesity health awareness

Self‐image and awareness	Denial of obesity
	Self‐neglect
	Self‐management

#### 3.2.1. Negative Perceptions About Obesity

##### 3.2.1.1. A Disease or Not a Disease?

The majority of the participants considered obesity a disease due to obesity being associated with the risk of other diseases and complications. Others considered it a disease due to its impact on appearance, mental health, and normal daily activities.

“Opens up the door to many other diseases and mental addiction to food” P47, male

“Because it affects the person, affects their energy and their ability to exercise and walk, and their appearance becomes unacceptable, and diseases increase” P50, female

Some participants stated that obesity is not contagious and therefore does not constitute a disease and relates to lifestyle and behavior, and there is no medication to combat it; therefore, it is within the patients’ remit to control.

“It’s not something that is contagious or needs medication to be treated. It’s a lifestyle and checking what the root cause from overeating.” P44, male

##### 3.2.1.2. Long‐Term Concerns

Participants also reflected on the long‐term impact of obesity and diabetes health struggles when they advance in their ages.

“To grow old and still be living with obesity and diabetes is a very daunting thought.” P17, female

##### 3.2.1.3. Factors Contributing to Obesity Development

The use of high insulin doses was also thought to have contributed to the development of obesity. Learning carb counting gave participants the freedom to eat what they liked and inject the correct amount of insulin units, and this has led to an increase in consumption and thus weight gain.

“Using insulin is a major factor, as well as having diabetes and hypoglycaemia.” P8, female

“[I developed obesity] because of carb‐counting skills and the ability to eat at any time” P39, male

#### 3.2.2. Poor Interface With Healthcare Professionals (HCPs)

##### 3.2.2.1. Diagnosis Deficits

Although all the participants were living with obesity, the findings indicated many were never officially diagnosed. They were offered lifestyle advice, but a conversation regarding obesity and management was not broached.

I was never diagnosed with obesity by my doctor. I was given advice on exercising to maintain my weight and prevent obesity.” P38, female

Another finding relating to diagnosis is the patient’s belief that they are not living with obesity, which may have dissuaded them from seeking a diagnosis and/or an intervention.

“I’m not obese, I am just slightly overweight and I’ve started exercising and have a diet plan.” P15, female

##### 3.2.2.2. Treatment Options

In regard to seeking treatment and participant preferences, the majority acknowledged that they sought treatment through medication followed by diet and exercise, and some suggested bariatric surgery. Some of the participants never sought treatment; this was attributed to a variety of factors, which include the following: believing that insulin treatment would stop them from losing weight and wanting to self‐manage obesity using exercise and nutrition without any external help.

“Because I don’t need it [treatment]. I can lose weight by doing workouts.” P49, female

##### 3.2.2.3. Role of Dietitians

Participants stated why they had never visited or stopped visiting their dietitian; these reasons include rigid and repetitive diet plans and instructions, no need for follow‐ups as they believe they know how to manage their diet after a few visits, easy accessibility of online diet plans, and inability to commit to a lifestyle.

“I know that dietitians have a traditional method for managing obesity and most people with diabetes don’t prefer being on diets.” P10, female

#### 3.2.3. Lack of Suggestions for Improving Obesity Management in T1D

##### 3.2.3.1. Position Diabetes Within Obesity Management

Many participants mentioned that it would be helpful if HCPs strive to increase awareness, position diabetes care within obesity management, and aim to reduce their insulin needs. In their efforts to manage their patients’ weight, HCPs should focus on improving their glycemic control and reducing their episodes of hyperglycemia and hypoglycemia.

“[HCPs] need to put in more efforts [in treating obesity] with increasing awareness about the effect of diabetes and insulin [on obesity] and how to exercise and avoid hypos.” P7, male

Furthermore, HCPs should endeavor to understand what challenges patients with T1D who have obesity encounter as they are already managing another chronic disease.

“[HCPs need to] understand the current burden of being a T1D and how much it consumes from his day and how stressful it is.” P47, male

##### 3.2.3.2. Focus on Mental Health

Participants asked for a more focused approach toward mental health, as this was perceived as lacking in their health care. Some participants reported feeling disheartened by weight gain shortly after periods of weight loss, adversely affecting their motivation.

“Obesity is very exhausting, especially when you lose the weight then regain it.” P7, male

Participants recommended that mental and physiological support was essential before embarking on obesity management, as they believe that it is necessary to be in the right mindset.

“A diagnosis with obesity and getting a patient motivated enough to fix it are two different things. The mind set of an obese person is pretty set in their ways.” P46, male

##### 3.2.3.3. Patient‐Centered Care

Participants believed that a patient‐centered approach toward obesity management was lacking. Participants suggested that HCPs should listen to and be mindful of patient concerns and ensure their mental health is taken into consideration.

The approach of HCPs to obesity management was highlighted as requiring some improvement. Participants stated that treatment and management options should be individualized to match the patients’ needs. Some participants recalled their own experiences with HCPs who did not appear interested in understanding the root cause of their obesity development, instead diving straight into treatment options.

“I think it’s the solutions provided, they don’t fit with the lifestyle. Very idealistic and does not work for everyone. Instead of just providing advice and suggestions, look at the person’s lifestyle and find solutions to fit that.” P44, male

Discussions surrounding obesity can also be enhanced by explaining obesity in easily understandable terms and engaging with the patient to further explain *why* obesity is a disease, how one can live with obesity, which complications can result from it, and the available treatment options. Obesity should be discussed gradually over visits until the patients’ trust is gained and they are ready for further discussion and treatment. HCPs should be firm but should never resort to “shock tactics,” which participants stated as having a negative impact; instead, focusing on health gains after weight loss is a better approach.

“By explaining what is obesity in a good way, by starting with what is it and how can we live with it and then finding a way to show its effects.” P50, female

##### 3.2.3.4. Improve Obesity Health Awareness

Improving obesity awareness was advocated by participants, with a special focus on the younger generations to prevent further problems from developing, particularly if there is a genetic disposition.

“Follow‐ups, follow‐ups, follow‐ups. And awareness to start from a young age.” P7, male

#### 3.2.4. Poor Self‐Image and Awareness

##### 3.2.4.1. Denial of Obesity

Some participants indicated that they were not living with obesity, as they did not perceive themselves as such. This led to them not seeking help or treatment. Instead, they attributed their weight gain to a variety of factors, such as married life, traveling, and the COVID pandemic.

“I don’t consider myself obese.” P20, male

##### 3.2.4.2. Self‐Management

Some participants stated that they were able to manage on their own and did not need a medical professional for an official diagnosis. Others focused on lifestyle measures for living well with their obesity. Some participants preferred to be self‐sufficient with their obesity management.

“I have determination [to lose weight] and I eat healthy.” participant who answered “does not matter” to obesity diagnosis question. (P23, female)

“I know I am [living with obesity] and I need to tackle it, dwelling on it won’t help.” participant who answered “does not matter” to obesity diagnosis question (P44, male)

## 4. Discussion

This study explored the complexities in patients with the diseases of obesity and T1D, shedding light on the interplay between body weight perceptions, contributing factors, and management challenges. The study’s quantitative analysis highlighted a discordance between participants’ perceived and actual body weight, emphasizing the psychological dimensions of living with both T1D and obesity. Despite all participants having a clinically measured BMI > 27 kg/m^2^, a substantial number perceived themselves as not having obesity but being overweight. Notably, one participant perceived their body weight as normal, although their actual BMI was 29.8 kg/m^2^.

In our study, participants’ preference to self‐identify as overweight instead of obese likely functioned as an adaptive coping mechanism in response to diabetes‐related distress. This strategy may preserve self‐esteem but can also reduce willingness to participate in structured weight management interventions. Similar to our study, a previous study reported widespread misperception of weight status among people with T2D, where many people labeled themselves as “overweight” rather than “obese” despite objective measures indicating obesity. Similar trends have been observed in nondiabetes patients where patients’ perception of having obesity was significantly lower than the actual prevalence of obesity [21, 22], This incongruence could be attributed to complex biopsychosocial factors [5], emphasizing the need to move beyond simplistic biomedical perspectives when addressing obesity in T1D. Although psychological buffering has been documented in other chronic illnesses, its role in T1D remains insufficiently examined [23]. Thus, these findings extend the literature by demonstrating the interaction between perceptual and emotional defenses and biomedical complexity, which shapes the lived experience of double disease.

The study’s quantitative findings suggested that patients believe lifestyle factors were the primary contributors to obesity in people with T1D, aligning with existing literature on the association between obesity and environmental factors, sedentary lifestyles, and dietary choices [15, 24]. Importantly, participants identified lifestyle as a more significant RR factor compared to other contributors [15], emphasizing the patients’ perception that they are responsible for having developed the disease of obesity. However, this perspective obscured the biological and iatrogenic factors contributing to weight gain in T1D, specifically hyperinsulinemia, glucose variability, and appetite dysregulation resulting from intensive insulin therapy [5, 9]. This duality acknowledging obesity as a “disease” echoed findings from qualitative research in nondiabetic populations [16].

Beyond lifestyle, a spectrum of contributing factors, including genetic predispositions, emotional states, societal and cultural pressures, and the side effects of medication, were also mentioned. This multifactorial landscape emphasized the need for individualized approaches, given the hesitance of patients regarding obesity as a disease and diminishing the biological contributors to the disease below their own lifestyle choices. Effective communication strategies are essential to reframe obesity as a complex, multifunctional disease and not a behavioral choice [19]. Our findings thus support the need for narrative‐based interventions that deconstruct stigma and align patient and provider understandings.

The qualitative arm of the study provided valuable insights into participants’ perceptions, worries, and experiences related to obesity and T1D. The identification of obesity as a disease by the majority of participants highlighted the recognition of its broader health implications, as the focus was on the biological complications of the disease and not the biological causes of the disease. However, the qualitative analysis revealed varied experiences of being diagnosed with obesity, with some participants expressing negative impacts on physical appearance, daily life, and mental health. Similar findings have been reported in the nondiabetes population [23]. The results suggested that preventive measures and supportive interventions should be implemented as effective strategies for addressing and coping with the psychological effects of obesity in individuals with T1D.

The participants’ concerns regarding diabetes‐related complications, increased blood glucose levels, and fatigue highlighted the interplay between obesity and T1D. In a similar study, there was more emphasis that T1D was also a barrier to engagement in healthy lifestyle behaviors’(18). These findings emphasized the importance of addressing obesity as a disease to mitigate its impact on T1D management. Similar findings have highlighted on the clinical support and practice guidelines that facilitate the dual management of weight and T1D in patients [17].

This study also unraveled gender disparities in perceptions of T1D as a barrier to obesity treatment, particularly among females. This gender‐specific nuance underscored the importance of tailoring interventions to address diverse needs and challenges experienced by individuals with T1D and obesity. Poor self‐image and denial of obesity were prevalent in the sample, highlighting the intersection of diabetes distress, depressive symptoms, and body dissatisfaction [8]. Women were especially likely to perceive T1D as a barrier to obesity treatment which is consistent with research indicating increased body image vulnerability and self‐stigma among women with T1D [18]. These results indicated the need for gender‐sensitive interventions that incorporate psychological and motivational frameworks into diabetes care.

Participants’ preferences for lifestyle measures as the primary weight loss option aligned with the current emphasis on behavioral interventions. Many people with T1D in Kuwait experience both diabetes distress and depressive symptoms. The co‐occurrence of both symptoms is associated with many sociodemographic and clinical factors [8]. We have also previously shown that eating disorders are common in people with T1D [25], but in this instance, they believed that insulin treatment would stop weight loss and that was not considered indicative of an eating disorder but rather the biological fact from patients’ own lived experience. However, this study identifies a need to improve awareness of obesity‐related complications and promote a healthy lifestyle, suggesting potential avenues for targeted interventions [26].

A significant proportion of participants sought guidance from dietitians for obesity treatment, with the majority finding dietary advice helpful. This highlighted the pivotal role of dietitians in the multidisciplinary approach to obesity management in T1D. However, the study also revealed a gap in understanding dietitians’ roles among a considerable number of participants, emphasizing the need for clearer communication and education regarding the potential benefits of dietitian involvement. These results are consistent with previous findings in adolescent and adult populations, which indicated that T1D frequently receives greater clinical attention than obesity [17, 18]. Our findings extended these observations by demonstrating that adults too perceive T1D itself as a barrier to receiving obesity treatment suggesting that healthcare systems inadvertently reinforce the separation of metabolic and weight‐focused care. Notably, dietitians were perceived as accessible but underutilized, with participants describing interactions as rigid and unsustainable. Similar critiques have been made internationally, highlighting the need for evolving dietetic models that integrate behavioral psychology and flexibility [25].

The study’s insights have implications for HCPs, policymakers, and researchers involved in diabetes care. Tailored interventions that address the multifaceted nature of obesity in T1D, accounting for individualized needs and psychosocial dimensions, are crucial. Additionally, efforts to bridge the awareness gap regarding obesity‐related complications and the role of dietitians could enhance the effectiveness of obesity management strategies. There is also growing literature on the prevalence of overweight and obesity issues; hence, there is an urgent need to develop evidence‐based weight management guidelines and interventions that address the concerns of T1D individuals [11].

Limitations of the study include the cross‐sectional nature, and future research may include longitudinal studies to track the trajectory of obesity in T1D, explore the impact of evolving treatment strategies, and investigate the effectiveness of interventions tailored to individual needs. More qualitative methods, such as interviews or focus group discussions, would have been included to complement the survey data and provide deeper insights into the lived experiences of individuals with obesity and T1D.

The relatively small number of participants should be seen within the context of qualitative research studies where formal power calculations are not a valid approach but rather saturation of themes is used to determine when to stop recruiting participants. Moreover, with the high prevalence of eating disorders with this study population, the study would have been strengthened by including other validated measures in addition to the survey developed. The questionnaire was used as part of the mixed methodology within the context of qualitative research; as such, it was a valuable qualitative research tool, but it will require both testing for validity and reliability. Validity refers to whether the results do represent what they are supposed to measure, and reliability refers to the consistency of a measure, that is, whether the results can be reproduced under the same conditions. Moreover, addressing gender‐specific challenges and perceptions can inform targeted interventions that optimize care for diverse populations within the T1D community.

## 5. Conclusion

This mixed‐methods study provided valuable insights into the complexities of patients with the diseases of obesity in T1D, emphasizing the need for a holistic understanding that goes beyond glycemic control. The findings highlighted the perceived multifactorial etiology of obesity, the psychological dimensions of living with both conditions, and the importance of patient‐centered, multidisciplinary approaches in managing this dual challenge.

### 5.1. Implications for Practice and Research

This study makes three main contributions to the existing research. First, it provides a rare empirical analysis of how individuals with T1D conceptualize obesity, demonstrating the continued prevalence of behavioral explanations even in the presence of biomedical understanding. Second, it identifies T1D as a perceived barrier to obesity treatment, an insight largely absent from previous research, which has predominantly focused on T2D populations. Third, it contextualizes these findings within a Middle Eastern setting, thereby contributing to global discourse by emphasizing the cultural and healthcare system factors that shape weight management.

Collectively, these findings support an emerging shift from glucose‐centric to person‐centric paradigms in T1D care. Integrating psychological support, stigma reduction, and flexible nutritional guidance within multidisciplinary teams may mitigate both obesity and diabetes‐related distress. Future research should utilize longitudinal and ethnographic methods to capture evolving self‐perceptions and to examine how integrated interventions influence engagement, metabolic outcomes, and quality of life. In a healthcare environment, this study highlights the importance of integrating patient perspectives. Effective obesity management in T1D necessitates attention to both quantitative outcomes and individual experiences.

**Nomenclature**
WHO: World Health Organization
T1D: Type 1 diabetes
T2D: Type 2 diabetes
DAFNE: Dose Adjustment for Normal Eating
BMI: Body mass index

## Disclosure

The communication reflects the author’s view, and neither the IMI nor the European Union, EFPIA, or any Associated Partners are responsible for any use that may be made of the information contained therein.

## Conflicts of Interest

Carel W. Le Roux has received personal fees from Boehringer Ingelheim, Eli Lilly, GI Dynamics, Gila Pharmaceuticals, Herbalife, Johnson & Johnson, Keyron, Novo Nordisk, and Zealand Pharma outside the submitted work. The other authors declare no conflicts of interest.

## Author Contributions

Ebaa Al Ozairi—conceptualization, study design, interpretation, and critical review and editing.

Dalal Alsaeed—study design, analysis, interpretation, and writing–original draft.

Alvin Mondoh—analysis, interpretation, and writing–original draft.

Etab Taghadom—data acquisition.

Mohammad Irshad—analysis, interpretation, and writing–original draft.

Dherar Alroudhan—data acquisition.

Jumana Al Kandari—data acquisition.

Werd Al‐Najim—conceptualization, study design, interpretation, validation, and critical review and editing.

Carel W. le Roux—conceptualization, study design, interpretation, validation, and critical review and editing.

## Funding

SOPHIA has received funding from the Innovative Medicines 2 Joint Undertaking under grant agreement No. 875534. This Joint Undertaking received support from the European Union’s Horizon 2020 research and innovation program and EFPIA and T1D Exchange, JDRF, and Obesity Action Coalition.

## Data Availability

The data that support the findings of this study are available upon request from the corresponding author. The data are not publicly available due to privacy or ethical restrictions.
